# Beyond traditional hypoxemia metrics: hypoxic burden as a predictor of cognitive dysfunction in sleep apnea

**DOI:** 10.1093/sleep/zsae292

**Published:** 2024-12-18

**Authors:** Aristotle G Leonhard, Vishesh K Kapur

**Affiliations:** The Division of Pulmonary, Critical Care, and Sleep Medicine, The University of Washington, Seattle, WA, USA; The Division of Pulmonary, Critical Care, and Sleep Medicine, The University of Washington, Seattle, WA, USA

Cognitive impairment is a particularly troublesome comorbidity of obstructive sleep apnea (OSA) that diminishes quality of life. OSA-related cognitive impairment commonly involves the domains of attention and vigilance, learning and memory, and executive function [1]. Excessive daytime sleepiness contributes to neurocognitive dysfunction, though the specific mechanisms of how OSA leads to cognitive deficits are still debated. Several potential causes have been proposed, including sleep fragmentation and the influence of cardiac and metabolic comorbidities. Among these, the impact of hypoxemia on brain regions involved in cognition, such as the prefrontal cortex and hippocampus, has emerged as a particularly compelling mechanism [1].

Metabolically active neuronal groups are particularly vulnerable to damage during hypoxia and reoxygenation events, leading to oxidative stress, inflammation, and endoplasmic reticulum stress responses [2]. Building on this pathway, epidemiologic studies have highlighted hypoxemia as a key exposure contributing to cognitive decline. For example, cross-sectional analyses from the Apnea Positive Pressure Long-term Efficacy Study (APPLES) indicated that hypoxemia measured as the percent of sleep time with oxygen saturation less than 85% (T85), rather than the apnea–hypopnea index by 4% desaturation criteria (AHI4%), was associated with adverse effects on motor and processing speed [3]. Longitudinal analyses from the Multicenter Outcomes of Sleep Disorders in Men (MrOS Sleep) Study found that for every 1% of time spent with an oxygen saturation less than 90% (T90), there was a 0.43 annualized decline in the mini-mental status exam score [4]. Interestingly, while an increase in the 3% oxygen desaturation index (ODI) corresponded to a worsening annualized score in the mini-mental status exam, the AHI with a 3% oxygen desaturation (AHI3%) did not meet statistical significance [4]. Findings from the HypnoLaus study with specific focus on elderly individuals showed that a lower mean oxygen saturation was associated with a greater decline in multiple domains of cognitive testing, while T90 was associated with a decline in processing speed and executive function [5]. In contrast, other studies have not shown a link between hypoxemia and cognitive decline; for example, in the Atherosclerosis Risk in Communities study, T90, AHI4%, and the arousal index were not associated with cognitive decline [6].

The inconsistency of associations across studies raises the question of what constitutes the optimal marker for exposure to hypoxemia. Metrics such as the T90 and T85 represent arbitrary thresholds that may be influenced by underlying cardiac and/or lung disease. The 3% and 4% ODI capture individual events of oxygen desaturation, but they do not account for the duration or the depth of desaturation, both of which potentially could impact cognitive impairment. To address these limitations, a recently developed metric, the sleep apnea-specific hypoxic burden (HB), seeks to better characterize the severity of hypoxemia. HB is calculated based on the area under the curve from baseline oxygen saturation before an event, thereby taking into account the depth and duration of the oxygen desaturation across events [7]. Of relevance to cognitive impairment, a recent study found that HB was associated with white matter hyperintensity volume (a marker of small vessel disease) as measured on magnetic resonance imaging, unlike the AHI4% which was not [8].

Amid growing interest in the role of hypoxic burden, Huang *et al*. provide new evidence in this edition of SLEEP linking HB to mild cognitive impairment (MCI), as assessed by the Montreal Cognitive Assessment (MoCA) [9]. Logistic regression models were used to assess the association between HB and MCI. The highest quartile of HB was found to be an important predictor of MCI, and the odds ratios (ORs) for MCI remained statistically significant in the fully adjusted model (OR 7.69 [95% CI 1.15–51.55]). This was also true for HB in REM (OR 8.87 [95% CI 1.22–64.34]) but not HB in NREM (OR 4.62 [95% CI 0.78–27.51]). A notable strength of this study was the analysis of other commonly used indicators of OSA severity and/or hypoxemia, such as the AHI by 3% desaturation or arousal criteria, ODI, T90, percent of sleep time with an oxygen saturation less than 88% (T88), minimum oxygen saturation, and mean oxygen saturation. The highest quartile of T90 (OR 7.08 [95% CI 1.26–39.83]), T88 (OR 7.50 [95% CI 1.26–44.79]), and mean oxygen saturation (OR 0.14 [95% CI 0.03–0.79]) had significant associations with MCI in models adjusted for age, gender, education, BMI, Epworth Sleepiness Scale, current smoking, and current alcohol. But they were no longer significant in the fully adjusted models that also included hypertension, diabetes, and low-frequency/high-frequency ratio of heart rate variability.

While Huang *et al*.’s study provides valuable insights, it is important to recognize its limitations. The cross-sectional study design and modest sample size (*N* = 116) from a single center warrant caution in interpreting the results. Larger longitudinal studies are needed to confirm these findings to ensure generalizability and provide stronger causal evidence. The adjustment for a number of potentially confounding factors is a strength, though there is a concern that some factors such as hypertension, diabetes, and heart rate variability may represent mediators of the impact of hypoxemia on cognitive impairment. If so, the fully adjusted models may underestimate the relationship between the hypoxemia measure and outcome.

These findings bolster prior literature suggesting hypoxemia as a pivotal exposure in OSA that impairs cognition [3–5]. Furthermore, they reinforce emerging evidence that hypoxemia during REM sleep has greater detrimental impact on cognitive function than hypoxemia during NREM sleep [10, 11]. Finally, they suggest that HB may be a more relevant measure of exposure to hypoxemia than traditional measures such as the ODI, T90, and T88 in predicting cognitive impairment.

Huang *et al*.’s findings contribute to the growing body of literature on the relationship between hypoxic burden and OSA-related outcomes [12–16]. In longitudinal cohorts, HB predicted cardiovascular disease-related mortality, heart failure, and stroke, but not cancer or venous thromboembolism (VTE). Compared to traditional measures of hypoxemia, HB was generally a superior predictor of risk when compared to the ODI. However, in some studies, T90 had comparable predictive ability for certain outcomes (such as stroke and major adverse cardiovascular events) and was a better predictor of cancer incidence and VTE [12–16].

OSA-related oxygen fluctuations occur in a variety of patterns across the night and differ between patients. The oximetry signal not only reflects hypoxemia exposure but also provides insights into the pathophysiology of sleep apnea [17]. The development and validation of area-based hypoxic measures such as the HB, which account for duration and depth of respiratory events, represent an important advance in helping us understand the impact of OSA on a variety of outcomes [12–14]. The current study suggests that HB may provide a more robust measure of the impact of hypoxemia on cognition in comparison to other commonly used measures in OSA. Given the complexity of the information provided by oximetry, a single metric may not be sufficient to fully capture the characteristics that explain the diversity of OSA-related outcomes among affected patients. This is suggested by the heterogeneity of findings from studies comparing measures of hypoxemia to a variety of outcomes in different populations. Further evaluation of the predictive capability of a range of conventional and novel hypoxemia metrics in diverse populations is needed across the range of outcomes among patients with OSA. Ultimately, this could guide clinicians to better use an array of oximetry metrics provided by sleep study data to personalize OSA care.

## Data Availability

No new data were generated or analyzed in support of this editorial.
